# The power of registries and radiostereometric analysis (RSA)

**DOI:** 10.2340/17453674.2024.41169

**Published:** 2025-01-07

Few new technologies have had such an impact on a clinical discipline as joint replacement has had on orthopedics. Suddenly, a previously incurable and incapacitating disease could be successfully treated by replacing major joints with metal and plastic, the results possibly lasting for decades. This new technology needed supervision and, in 1975, the Swedish Knee Arthroplasty Register (SKAR) was launched with the explicit purpose of identifying potential faulty designs and operative procedures [1]. Aseptic mechanical loosening of tibial components was soon identified as the most common major complication in knee arthroplasty. The etiology of such mechanical loosening was envisaged as analogous to “breaking through the ice”—a traumatic episode of failed fixation at the implant–bone interface [2]. The successful SKAR concept was soon followed by the Swedish Hip Arthroplasty Registry and the appealing concept of national registries that yielded large numbers and reflected results from entire surgical communities has now become globally ubiquitous.

In 1974, Göran Selvik presented his Roentgen Stereophotogrammetric Analysis (RSA) research methodology [3,4] and RSA of component migration was soon applied to arthroplasty research. It was eventually realized, for example, that mechanical loosening was not caused by sudden traumatic ruptures at the soft tissue interfaces but rather represented biological processes in the bone–implant interface that started early and sometimes reached clinical significance only after many years [5]. RSA of component migration for 1–2 years became a prognostic tool for predicting clinical aseptic loosening for some fixations [5,6].

Hence, as long as 50 years ago, 2 powerful technologies were available to assess arthroplasties in such a way that as few patients as possible were put at risk when new implant designs or surgical procedures were introduced.

Both national registries and RSA have proven to be hugely successful. A simple search on PubMed will yield 500+ papers on RSA (RSA NOT reverse shoulder AND joint prostheses) and a similar search on registries (joint registry AND orthopedics) will yield 3000+ hits. Joint registries, however, have focused on 5–10-year survivorship of huge numbers of patients and RSA has been plagued with different potential threshold values for different implants/fixations. Certainly, a number of faulty prosthetic designs or procedures have been withdrawn but often only after conventional clinical observation and RSA corroboration subsequently. For example, Boneloc cement showed early clinical failure, which was only later confirmed by RSA [7]. The Charley Elite Plus was introduced in 1993. In 2006 there were some alarming RSA reports on fixation [8] and later the importance of good cementing techniques was emphasized [9]. The ill-fated ASR hip, later withdrawn for ALVAL (i.e., aseptic lymphocyte-dominant vasculitis-associated lesion) reactions, was interpreted by RSA to behave benignly regarding fixation [10]. With the increased tightening of regulatory requirements, notably the new Medical Device Regulation (MDR) in Europe with demands for clinical proof of concept already at certification [11], both conventional RSA and registry data may be insufficient; relevant results need at least 2–5 years to be decisive.

*Acta orthopaedica* is now presenting a paper by Puijk et al., in which the results from 2 parallel meta-analyses, 1 on arthroplasty registries and 1 on RSA, were combined [12]. None of the included studies (n = 285) contained both kinds of data but they were linked by their combination of prosthetic design, mode of fixation, and type of bearing, resulting in 185,376 observations for statistical analysis. This work represents a culmination of decades of arthroplasty registries and RSA research efforts to establish threshold values for initial and short-term RSA migration. Previously, in 2012, it was suggested that 12-month migration could be enough and in 2023 the same authors suggested that even a 6-month threshold could be of clinical significance [13,14]. In the current article the authors depart from the 2012 article and find that, since then, an additional 46 RSA studies and 127 register studies could be identified representing 415 new registry–RSA pairs. Using the 12-month migration thresholds, 2 (0.5%) of the new entries were miscategorized, i.e., they were classified as being “at risk” despite good migration data (n = 1) or good RSA data (n = 1). The authors conclude that 12-month migration data can be used to make a clear statement as to future clinical performance and further suggest that 6-month RSA thresholds that are only slightly less stringent may suffice.

The value of such a situation is hard to overestimate. Again, the new MDR legislation in Europe now obliges substantial clinical documentation to receive a CE certificate for any new device. These new demands may put an excessive burden on manufacturers, which may opt to remove devices from the market. The new data by Puijk et al. will allow sufficient documentation to be obtained in a short time (6–12 months) and with a minimum of patients at risk with relevant and agreed thresholds. These remarkable efforts to combine decades of arthroplasty registry and RSA data on hundreds of thousands of patients arrives at a time when more facile RSA techniques with computed tomography [15,16] have reached a resolution similar to the orthodox tantalum marker RSA. This will further enhance patient safety while allowing for necessary evolution and introduction of new arthroplasty technologies.


**Michael Dunbar** *Professor of Surgery, Dalhousie University, Halifax, Nova Scotia, Canada.* *Email: michael.dunbar@dal.ca*
**Leif Ryd** *Former Professor of Orthopaedics, CLINTEC, Karolinska Institute, Stockholm, Sweden.* *Email: leif@leryd.se*

